# The neighborhood food environment: sources of historical data on retail food stores

**DOI:** 10.1186/1479-5868-3-15

**Published:** 2006-07-17

**Authors:** May C Wang, Alma A Gonzalez, Lorrene D Ritchie, Marilyn A Winkleby

**Affiliations:** 1 School of Public Health, University of California at Berkeley, CA, USA; 2 Center for Weight & Health, College of Natural Resources & School of Public Health, University of California at Berkeley, CA, USA; 3 Stanford Prevention Research Center, Stanford University School of Medicine, USA

## Abstract

With the rapidly increasing prevalence of obesity in the United States, and the minimal success of education-based interventions, there is growing interest in understanding the role of the neighborhood food environment in determining dietary behavior. This study, as part of a larger study, identifies historical data on retail food stores, evaluates strengths and limitations of the data for research, and assesses the comparability of historical retail food store data from a government and a commercial source.

Five government and commercial listings of retail food stores were identified. The California State Board of Equalization (SBOE) database was selected and then compared to telephone business directory listings. The Spearman's correlation coefficient was used to assess the congruency of food store counts per census tract between the SBOE and telephone business directory databases. The setting was four cities in Northern California, 1979–1990.

The SBOE and telephone business directory databases listed 127 and 351 retail food stores, respectively. The SBOE listed 36 stores not listed by the telephone business directories, while the telephone business directories listed 260 stores not listed by the SBOE. Spearman's correlation coefficients between estimates of stores per census tract made from the SBOE listings and those made from the telephone business directory listings were approximately 0.5 (*p *< .0001) for the types of stores studied (chain supermarkets, small grocery stores, and chain convenience markets). We conclude that, depending on the specific aims of the study, caution and considerable effort must be exercised in using and applying historical data on retail food stores.

## Short paper

With the increasing prevalence of obesity in the United States [1], and the minimal success of education-based interventions [2,3], there has been growing interest in investigating the role of the neighborhood food environment in influencing dietary behavior [4-8]. Such an investigation requires an operational definition of the neighborhood food environment, and methods for acquiring the relevant data.

The neighborhood food environment can be operationally defined by counts of various types of retail food stores or restaurants, which have been used to indicate the types of foods that are readily available within a neighborhood [7-10]. These counts may be expressed relative to geographic area (e.g. number of supermarkets per square mile) or in absolute terms (e.g. number of supermarkets per neighborhood). Another way to indicate the accessibility of various types of foods in a neighborhood is proximity or the closest distance to a particular type of food store from a participant's residence [8]; such a method has been used to indicate accessibility of other health-related goods and services such as alcohol [11,12]. Neighborhoods may be defined using census tract boundaries [13-15].

The primary aim of this paper is to describe the methodological challenges of measuring past neighborhood food environment. This effort was conducted as part of a larger study aiming to examine the associations between the neighborhood food environment and obesity risk using epidemiologic data previously collected from over 8000 men and women, aged 18–74 years, by the Stanford Heart Disease Prevention Program (SHDPP) between 1979 and 1990 [16]. In particular we will identify historical sources of retail food store data, assess the strengths and limitations of these data sources and, evaluate the comparability of two selected data sources.

### Identification of databases

We searched the internet, and contacted health, agriculture and business licensing government departments to determine potential databases relevant to the aims of our larger study. In particular, we sought databases that were likely to: (a) contain store name, street address, and dates of operation; (b) provide information indicating the type of food store (supermarket, convenience store, small grocery store, etc.), and size of operations [such as annual sales volume, number of cash registers, store area (square footage)]; and (c) include small grocery stores. In addition, for the purposes of our larger study, which involved a retrospective cohort [11,12,17], we required that data be available for the years, 1979–1990.

Information on food store type was needed to indicate the availability of healthy or unhealthy foods. Since these data were historical, we did not have the opportunity to observationally assess if the stores carried healthy or unhealthy foods. However, a few studies have observed that in the United States, supermarkets tend to carry healthy foods, while small grocery stores ('corner markets') and convenience stores are less likely to carry healthy foods, especially fresh produce [18,19]. Information on the size of operations was considered helpful for distinguishing small grocery stores from larger stores especially those that were independent and did not belong to a corporate chain.

We identified five databases, two from government sources (a city business licensing department, and the State Board of Equalization, SBOE) and three from commercial sources (Dun & Bradstreet, Trade Dimensions, and the telephone business directory).

### Strengths and limitations of the identified data sources

Strengths and limitations of these five databases are summarized in Table 1. In general, all government databases provide listings of stores by name and address. The SBOE maintained records of the initial date of application for a license or permit and subsequent dates of active renewal, allowing for the years of operation to be determined. Small grocery stores were generally included in government databases.

**Table 1:** Government and commercial retail food store data sources

Potential data source	Data available for the 1980s	Dates of operation	How often information is updated	Store name and address	Indicator of scale of operations^b^	Some information for classifying food store as supermarket, etc.	Include small grocery stores
GOVERNMENT SOURCES							
Local business licensing agencies	Yes^c^	Yes	When licenses are renewed	Yes	No	Yes	**Yes**
State Board of Equalization^d ^(California)	**Yes**	Can be derived from multiple renewal records	When permits are renewed	Yes	No	Yes	**Only those that carry taxable items^e^**

COMMERCIAL SOURCES							
Dun & Bradstreet^f^	Yes	Yes	Semi-annually	Yes	Yes^g^	Yes	Not usually
Trade Dimensions^h^	Yes	Yes	Annually or semi-annually	Yes	Yes	Yes	Stores with annual sales volume of <$0.5 m are not usually included
Yellow Pages	**Yes**	No	Updated only when advertisement contract is renewed	Address sometimes not available	No	Yes	**Usually**

^a^Information presented here was obtained in 2002–2003 for data relevant to 1979–1990.

^b^Indicators of scale of operations included annual sales volume, number of employees, number of cash registers, and store area (square footage).

^c^Only one city agency (Modesto) was able to retrieve data for 1979–1990.

^d^The California State Board of Equalization (SBOE) is responsible for collecting and allocating sales and use taxes from all businesses that sell taxable goods , and has records of all retail food stores except those that sell only non-taxable items such as fresh meat, produce and dairy. The data were obtained from Merlin Information Services (Kalispell, MT), a private vendor of national and California-specific public record information.

^e^All stores that sell taxable items are required by law to apply for a permit but compliance is not 100%.

^f^Dun & Bradstreet (Short Hills, NJ) is an established organization that maintains one of the most comprehensive business information databases in North America.

^g^Data for chain stores are available only at the corporate level, but not at the store level.

^h^Trade Dimensions (Wilton, CT) gathers data on the retail food industry and provides marketing information to organizations such as the Food Marketing Institute and Progressive Grocer.

Commercial databases that served to provide business related information, such as Dun & Bradstreet, and Trade Dimensions, were updated about once every 6–12 months, and provided indicators of the size of store operations. Dun and Bradstreet's information on size of operations was not available at the store level for chain stores. (For example, sales volume estimates were available for Safeway Corporation but not for a Safeway store at a specific location.) Small retail food stores with annual sales volume of less than $500,000 were generally not included in these two databases.

The telephone business directories were likely to include small grocery stores. However, their store listings were problematic for our purposes. Specifically, store addresses sometimes reflected the address of the headquarters office rather than the physical location of the store. Further, stores located in the same building were often listed using the same street address (without showing a suite number), making it difficult to decide if they were separate stores or the same store listed under different names.

### Comparability of databases

Since the SBOE and telephone business directory databases were the only sources that included listings of small grocery stores, they were selected for comparison. For the purposes of our larger study, we created datasets that contained records of stores that were open for any period of time during the years relevant to the data gathered by the SHDPP (1979–1990); the time period for which the store was open was recorded to subsequently allow for the store data to be properly matched to the year in which SHDPP participants were examined.

To assess the comparability of the two selected datasets, we matched records to determine the number of stores in one database that were also found in the other for the years 1979–1990. We further compared the store counts per census tract estimated by the two databases, and calculated the percentage of census tracts with similar store counts per census tract, derived from the SBOE database and the telephone business directory listings. We conducted these analyses for three major store types: supermarkets, small grocery stores, and chain convenience stores. (Other food store types were too few in number to allow for a meaningful analysis.) These stores were classified using store categories and definitions developed by the North American Industry Classification System , and the Food Marketing Institute :

• A supermarket is any self-service grocery store that generates an annual sales volume of >$2 million.

• A small grocery store/market is an independently owned store that sells beverages, tobacco, and a limited selection of convenience foods (including ethnic markets).

• A convenience store is any self-service grocery store that offers a limited line of high-convenience items; it is usually open long hours and provides easy access.

The SBOE and telephone business directory databases listed 127 and 351 retail food stores, respectively. The SBOE listed 36 stores not listed by the telephone business directories (28%), while the telephone business directories listed 260 stores not listed by the SBOE (74%). These additional stores listed in one database but not in the other were almost equally distributed among chain supermarkets, small grocery stores, and chain convenience markets. These data suggest that retail food store counts derived from the SBOE were likely to be underestimated; SBOE listings omitted stores that did not sell any taxable goods (in general, only a few food products such as hot-prepared foods and certain beverages, are taxable) and stores that failed to get a tax permit.

In contrast, retail food store counts derived from the telephone business directories were likely to be overestimated. The telephone business directories sometimes listed the same store multiple times under different categories, or under different names. Inquiries of telephone business directory staff revealed that any business with a valid phone number could pay to be listed under any number of categories or names, and for more than one year. This meant that telephone business directories might include stores that had recently closed. As a result, telephone business directories almost always gave a considerably higher count of stores than the SBOE database.

We also compared the counts of the various types of food stores per census tract, derived from both data sources (SBOE and telephone business directories). We found that 95%, 67% and 81% of the 84 census tracts in our study had counts of supermarkets, small grocery stores, and chain convenience stores respectively, that were identical or did not differ by more than one store. Identical agreement between estimates ranged from 39% for chain convenience stores to 57% for chain supermarkets. The correlation between SBOE- and telephone business directory-derived store counts per census tract was moderate (Spearman's correlation coefficient = 0.5, p < .0001) for all three types of stores studied.

To our knowledge, this is the first study to address the methodological challenges of measuring the neighborhood food environment in the United States. The following limitations to the applicability of our findings should be noted. Our findings are specific to information for the years 1979–1990. Investigations requiring current data will have access to more retail food store databases and also have the opportunity to interview store managers, and directly assess the quality and affordability of foods available in the stores. This paper does not include a discussion of sources of data on eating places. Finally, we did not examine all types of stores. Medium-sized independent supermarkets, and stores that specialized in produce, meat, seafood, etc. were too few in number to be meaningfully examined. Also, we did not examine ethnic markets separately from other small grocery stores. Ethnic markets may be more likely to carry fruits and vegetables than other small grocery stores. Qualitative data gathered by one of the authors (AG), from a socio-economically diverse group of 28 women suggest that ethnic markets, but not other small grocery stores, are perceived as predominant sources of quality and affordable fresh produce.

We recommend that researchers needing to use secondary data sources of retail food stores carefully evaluate the appropriateness of use of the databases. Studies using *past *secondary data sources should at the least, understand and recognize the limitations of historical databases, which suffer from the lack of a 'gold standard'. Studies using *current *secondary data sources could benefit from an effort to assess the validity of these sources through observational techniques.

## Competing interests

The author(s) declare that they have no competing interests.

## Authors' contributions

MCW conceived of and led the study, supervised and performed data analysis, and drafted the manuscript. AAG sought out the data sources, coordinated data collection, and contributed to the manuscript. LDR assisted with data interpretation and helped to draft the manuscript. MAW provided guidance on the study design, and data analysis and interpretation, and contributed to the manuscript. All authors read and approved the final manuscript.
